# Supplementary material for: Deletion of wfs1 Impairs Oligodendrocyte Precursor Cells Dorsal Distribution and Myelination Through the wfs1-hmgcs1 Axis in Zebrafish
Source: Biology (Basel). 2026 Aug 16;15(16):1402. doi: 10.3390/biology15161402 (PMC13510151; doi:10.3390/biology15161402)
Supplement: Supplementary file 1 [file biology-15-01402-s001.zip › File S1-Transcriptome sequencing data and differentially expressed genes (DEGs) in wfs1-- zebrafish at 5 dpf/2.KEGG/wfs1KOvsWT/ALL/src/dre03430.html]

dre03430


- dre:568323

- Up regulated genes

568323\_1(1.8296241908303)

- Down regulated genes

568323(-6.68771578830024)

- dre:556995

- Up regulated genes

556995\_1(2.4821943051154)

- Down regulated genes

556995\_2(-1.79188203078216)

- dre:565400

- Up regulated genes

565400(3.2508117513811) 565400\_1(1.14977126474414)

- dre:565400

- Up regulated genes

565400(3.2508117513811) 565400\_1(1.14977126474414)

Close
